# First record of the tetrablemmid armoured spiders (Araneae, Tetrablemmidae) from Xizang, China, with description of a new species

**DOI:** 10.3897/BDJ.12.e143503

**Published:** 2024-12-12

**Authors:** Yang Zhou, Xiaoqing Zhang, Yanfeng Tong

**Affiliations:** 1 College of Life Science, Shenyang Normal University, Shenyang 110034, China College of Life Science, Shenyang Normal University Shenyang 110034 China

**Keywords:** Distribution, morphology, new record, taxonomy

## Abstract

**Background:**

The spider genus *Choiroblemma* Bourne, 1980 of the family Tetrablemmidae currently contains two species, *C.bengalense* Bourne, 1980 and *C.rhinoxunum* Bourne, 1980. Both species are known from West Bengal, India.

**New information:**

A new species of *Choiroblemma* is described from Xizang, China, *Choiroblemmametok* sp. nov. (♂♀). Morphological descriptions, photomicroscopy images of the new species and a distribution map of *Choiroblemma* species are given.

## Introduction

The family Tetrablemmidae O. Pickard-Cambridge, 1873, commonly known as tetrablemmid armoured spiders, currently comprises 152 species in 27 genera (WSC 2024). Tetrablemmids are very small to medium-sized, cryptic, haplogyne spiders (Jocqué and Dippenaar-Schoeman 2006). They are mainly distributed in the tropical and subtropical regions where they are found in leaf litter, soil and in caves (Burger et al. 2010). In China, 23 species in eight genera have been recorded. Of these, seven species are from Hainan, five from Guangdong, four from Guangxi, four from Yunnan, one from Chongqing, one from both Chongqing and Guizhou and one from Taiwan. (Tong and Li 2008, Lin and Li 2010, Lin and Li 2014, Ballarin et al. 2021, He and Lin 2021, Cheng et al. 2022, Fu et al. 2022, Lin et al. 2022, He et al. 2024).

The genus *Choiroblemma* was established by Bourne, based on two species from West Bengal, India. It is diagnosed by the fine embolus and robust conductor sclerite of male palp and the sclerotised ridges on the pre-anal plate of the female (Bourne 1980). Examination of tetrablemmid specimens preserved in Hebei University (Baoding, China) led to the finding of one new species of *Choiroblemma*. It is the third species of the genus and the first discovery of the tetrablemmid armoured spiders in Xizang, China. In this paper, a new species of *Choiroblemma*, collected from Xizang, is described.

## Materials and methods

Specimens were examined using a Leica M205 C stereomicroscope. Fine details were studied under an Olympus BX51 compound microscope. The endogyne was cleared in lactic acid. Photomicroscope images were taken with a Canon EOS 750D zoom digital camera (24.2 megapixels) mounted on an Olympus BX51 compound microscope. Raw photos were first stacked with Helicon Focus v. 8.2.0 to obtain the composite images, which were then processed in Adobe Photoshop CC 2020. All measurements were taken using an Olympus BX51 compound microscope and are in millimetres. Terms and abbreviations used in the text and figures follow Cheng et al. (2022). Type material is deposited in the Shenyang Normal University (SYNU) in Shenyang, Liaoning Province, China (curator: Yanfeng Tong).

## Taxon treatments

### Choiroblemma metok sp. nov.

521A90F5-75A6-5C13-B183-49E3DEC7A48A

F9CD3EBD-625F-41EF-8558-C444AD948032

#### Materials

- **Type status:** Holotype. **Occurrence:** recordedBy: Panlong Wu; individualCount: 1; sex: male; lifeStage: adult; occurrenceID: DC568B4F-4B5F-5C52-B2B8-6E19CAB09EB5; **Taxon:** order: Araneae; family: Tetrablemmidae; genus: Choiroblemma; specificEpithet: *metok*; **Location:** country: China; stateProvince: Xizang; county: Nyingchi City, Metok County; verbatimCoordinates: 29°19′30″N, 95°19′59″E; **Identification:** identifiedBy: Yanfeng Tong; **Event:** eventDate: 21 September 2013; **Record Level:** institutionCode: SYNU-1379
- **Type status:** Paratype. **Occurrence:** recordedBy: Panlong Wu; individualCount: 1; sex: female; lifeStage: adult; occurrenceID: C59EA94F-9374-5001-A7B0-3902049CDCE1; **Taxon:** order: Araneae; family: Tetrablemmidae; genus: Choiroblemma; specificEpithet: *metok*; **Location:** country: China; stateProvince: Xizang; county: Nyingchi City, Metok County; verbatimCoordinates: 29°19′30″N, 95°19′59″E; **Identification:** identifiedBy: Yanfeng Tong; **Event:** eventDate: 21 September 2013; **Record Level:** institutionCode: SYNU-1380
- **Type status:** Paratype. **Occurrence:** recordedBy: Zhizhong Gao; individualCount: 5; sex: 1 male, 4 females; lifeStage: adult; occurrenceID: 7572C843-6313-5D55-93A0-100E837D9D38; **Taxon:** order: Araneae; family: Tetrablemmidae; genus: Choiroblemma; specificEpithet: *metok*; **Location:** country: China; stateProvince: Xizang; county: Nyingchi City, Metok County; locality: hills behind the Petrochina Motuo gas station; verbatimCoordinates: 29°19′46″N, 95°20′32″E; **Identification:** identifiedBy: Yanfeng Tong; **Event:** eventDate: 22 September 2013; **Record Level:** institutionID: SYNU-1374-1378
- **Type status:** Paratype. **Occurrence:** recordedBy: Yejie Lin; individualCount: 2; sex: male; lifeStage: adult; occurrenceID: C376BB62-898E-50BC-88A1-44B89FC39352; **Taxon:** order: Araneae; family: Tetrablemmidae; genus: Choiroblemma; specificEpithet: *metok*; **Location:** country: China; stateProvince: Xizang; county: Nyingchi City, Metok County; locality: Yadang Village; verbatimCoordinates: 29°20′36″N, 95°20′48″E; **Identification:** identifiedBy: Yanfeng Tong; **Event:** eventDate: 6 August 2013; **Record Level:** institutionID: SYNU-F-091-092

#### Description

**Figure 1.**
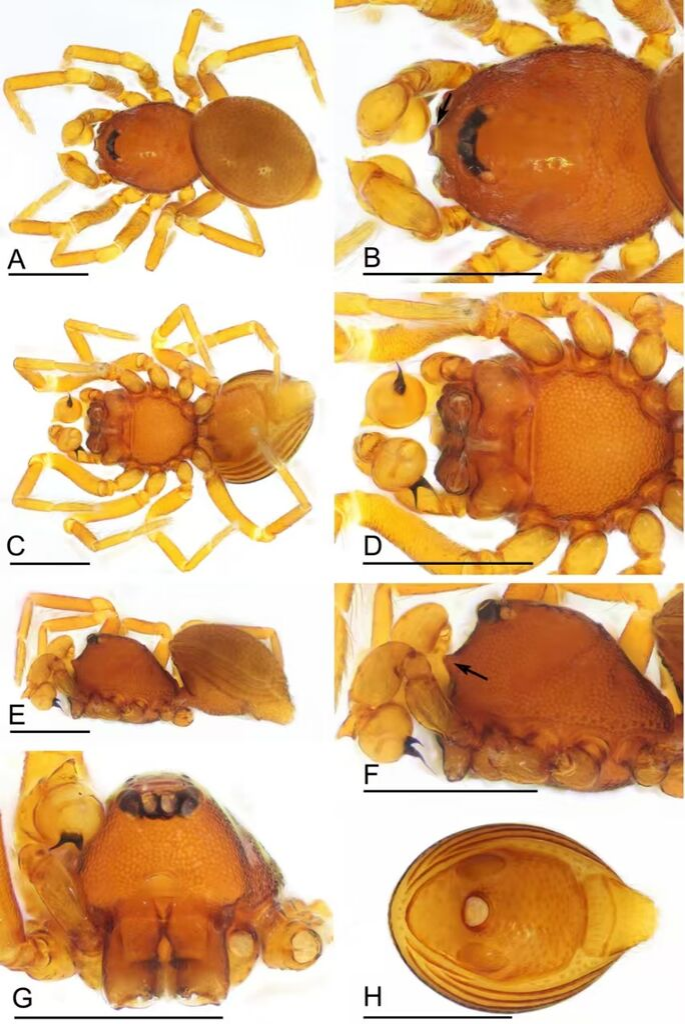
*Choiroblemmametok* sp. nov., male holotype. **A** habitus, dorsal view; **B** prosoma, dorsal view; **C** habitus, ventral view; **D** prosoma, ventral view; **E** habitus, lateral view; **F** prosoma, lateral view; **G** prosoma, anterior view; **H** abdomen, ventral view. Arrows show the snout-like protuberance in Figs. B and F. Scale bars: 0.4 mm.

Male (holotype). Colouration: body orange brown, legs yellowish. Habitus as in Fig. 1A, C and E.

Measurements: total length 1.13; carapace 0.55 long, 0.47 wide, 0.29 high; abdomen 0.73 long, 0.52 wide, 0.47 high; clypeus 0.16 high; sternum 0.32 long, 0.35 wide. Length of legs: I 1.30 (0.42, 0.14, 0.32, 0.21, 0.21); II 1.16 (0.38, 0.14, 0.26, 0.19, 0.19); III 0.99 (0.28, 0.13, 0.22, 0.20, 0.16); IV 1.37 (0.42, 0.14, 0.36, 0.24, 0.21).

Carapace reticulated, margin with small denticles; cephalic region slightly sloped in lateral view; eyes in three distinct pairs; clypeus very high, with distinct snout-like protrusion (Fig. 1B and F); chelicerae frontally with basally wide, conical horn, distally with well-developed prong (Fig. 3E); sternum with sparse setae, reticulated (Fig. 1D). Opisthosoma oval; dorsal scutum large, oval, glossy, densely pitted, covered with fine setae; pulmonary plate glossy, with pits for setae; pre-anal plate much wider than postgenital plate. Legs unmodified.

Palp (Fig. 3A–D): femur and tibia slightly swollen; cymbium with a conical apophysis; bulb subglobular; the embolus very thin and needle-like, conductor sclerite short, nearly half length of the embolus.

**Figure 2.**
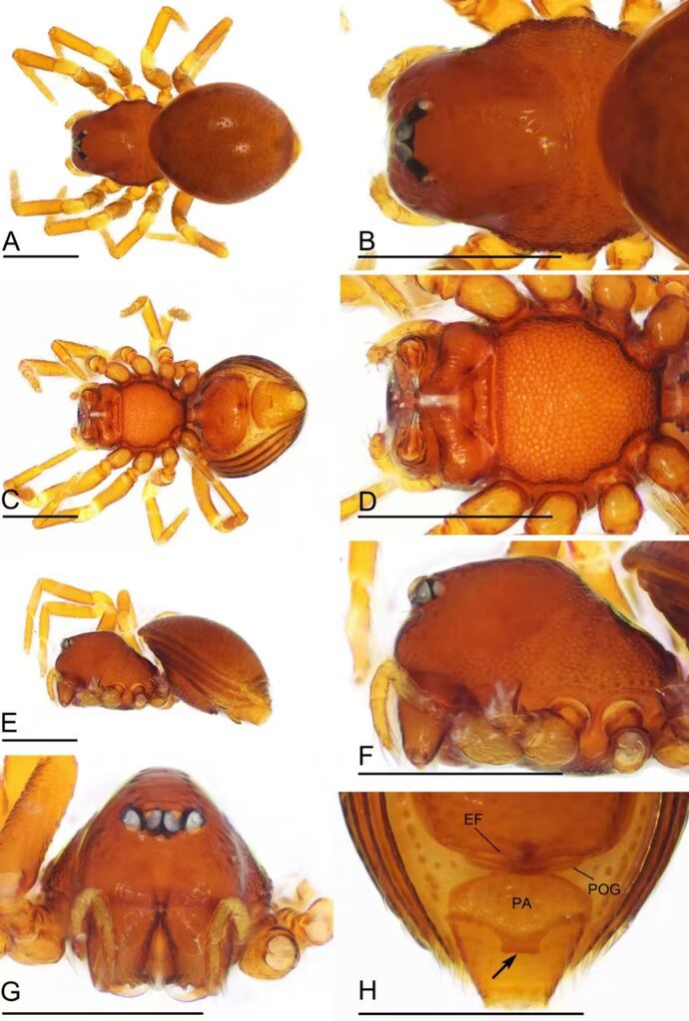
*Choiroblemmametok* sp. nov., female paratype (SYNU-1380). **A** habitus, dorsal view; **B** prosoma, dorsal view; **C** habitus, ventral view; **D** prosoma, ventral view; **E** habitus, lateral view; **F** prosoma, lateral view; **G** prosoma, anterior view; **H** genital area, ventral view, arrow shows the extension. Abbreviations: EF = epigynal fold; PA = pre-anal plate; POG = postgenital plate. Scale bars: 0.4 mm.

**Female** (SYNU-1380). As in male, except as noted. Habitus as in Fig. 2A, C and E.

Measurements: total length 1.23; carapace 0.50 long, 0.47 wide, 0.27 high; abdomen 0.83 long, 0.65 wide, 0.48 high; clypeus 0.13 high; sternum 0.34 long, 0.36 wide. Length of legs: I 1.25 (0.41, 0.15, 0.29, 0.20, 0.20); II 1.12 (0.41, 0.14, 0.21, 0.18, 0.18); III 1.00 (0.30, 0.13, 0.22, 0.18, 0.17); IV 1.42 (0.45, 0.16, 0.35, 0.25, 0.21).

Cephalic region slightly elevated in lateral view; clypeus without the snout-like protuberance; chelicerae horn absent (Fig. 2E and G).

Abdomen: epigynal fold wide; pre-anal plate with a rectangular extension on posterior margin (Fig. 2H).

**Figure 3.**
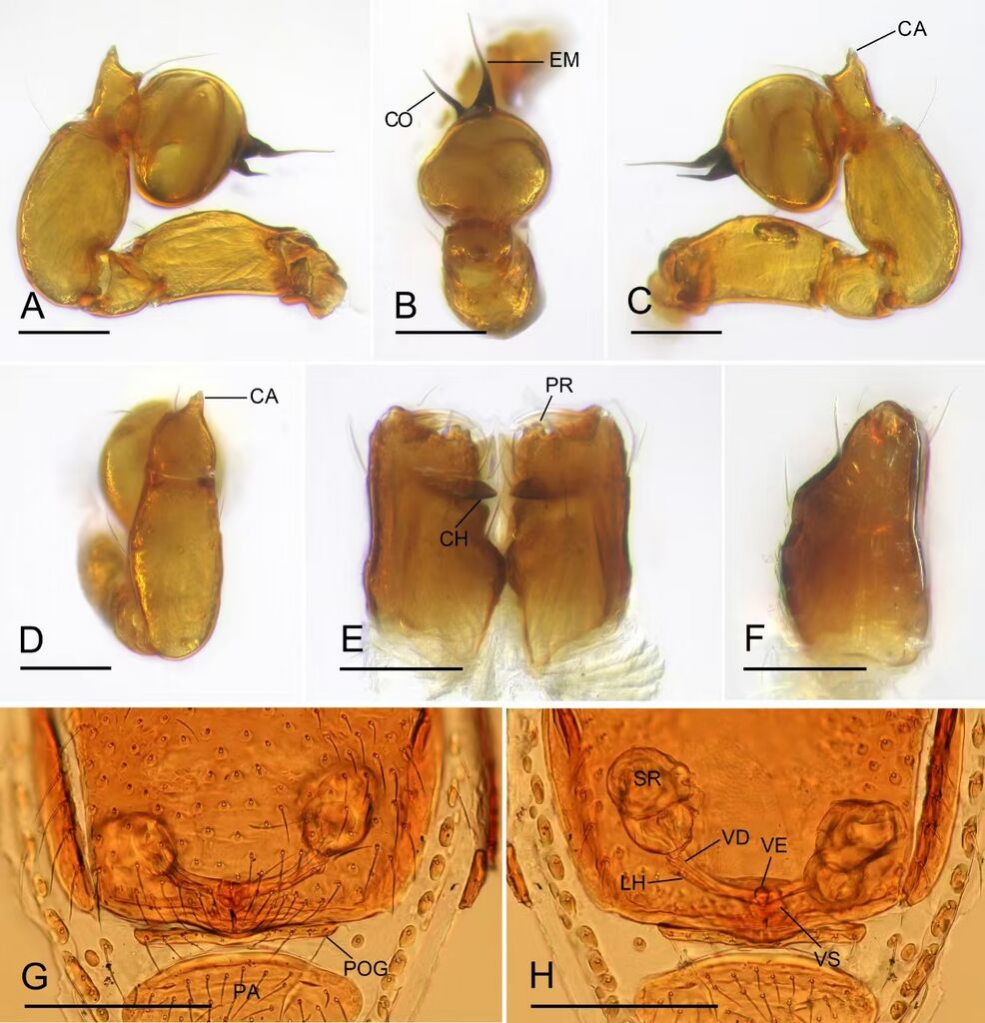
*Choiroblemmametok* sp. nov. **A** male left palp, prolateral view; **B** male left palp, distal view; **C** male left palp, retrolateral view; **D** left palpl tibia and cymbium, dorsal view; **E** male chelicerae, anterior view; **F** male chelicerae, lateral view; **G** female genital area, ventral view; **H** female genital area, dorsal view. Abbreviations: CA = conical apophysis; CH = cheliceral horn; CO = conductor; EM = embolus; LH = lateral horn; PR = prong; PA = pre-anal plate; POG = postgenital plate; SR = seminal receptaculum; VD = vulval duct; VE = vestibule; VS = vulval stem. Scale bars: 0.1 mm (A–F); 0.2 mm (G, H).

Genitalia: a very thin duct running into a small vestibule; vulval stem wide, strongly sclerotised; lateral horns long, slightly sclerotised, supporting the base of vulval ducts of seminal receptaculum (Fig. 3H).

#### Diagnosis

This new species is similar to *Choiroblemmabengalense* and *Choiroblemmarhinoxunum* (Bourne 1980: figs. 31–44) in the snout-like protrusion of clypeus of male and the thin embolus, but can be distinguished by the thin and short conductor (Fig. 3A–C), vs. wide and robust, the conical apophysis of male palpal cymbium (Fig. 3C and D) vs. lacking, the distally prong of male chelicerae (Fig. 3E) vs. lacking and the extension of female pre-anal plate on posterior margin (Fig. 2H) vs. on the middle area.

#### Etymology

The specific name is a noun in apposition taken from the type locality.

#### Distribution

**Figure 4.**
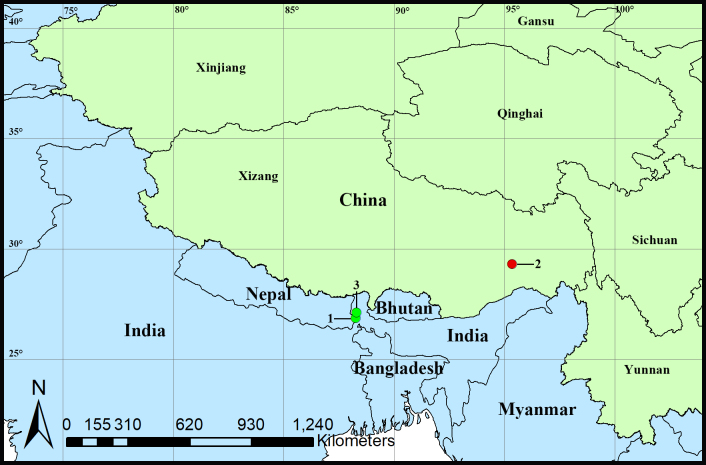
Distribution records of *Choiroblemma* species from China and India, green circles represent two known species, red circle indicates the new species. 1. *Choiroblemmabengalense* Bourne, 1980; 2. *Choiroblemmametok* sp. nov.; 3. *Choiroblemmarhinoxunum* Bourne, 1980.

Known only from the type locality (Fig. 4).

## Supplementary Material

XML Treatment for Choiroblemma metok
